# Dual contraceptive use and associated factors among women aged 15-49 years on antiretroviral therapy in Kayonza District, Rwanda: a cross-sectional study

**DOI:** 10.11604/pamj.2022.42.45.33460

**Published:** 2022-05-17

**Authors:** Jean Nepomuscene Renzaho, Erigene Rutayisire

**Affiliations:** 1 Department of Public Health, Mount Kenya University-Rwanda, Kicukiro District, Kigali, Rwanda

**Keywords:** Dual contraception, women in reproductive age, antiretroviral therapy

## Abstract

**Introduction:**

globally, 600,000 women died of complications due to pregnancies among more than 2 million women on antiretroviral treatment who get pregnant every year due to low utilization of dual contraception and unsafe sex. The failure rate of preventing of mother-to-child transmission of HIV/AIDS (PMTCT) in Rwanda was 2% in 2019. In Rwanda, there was no research done and published on this topic. To fill the gap, the study aimed to assess the use of dual contraceptives and associated factors among women aged 15-49 years on antiretroviral (ART) in Kayonza District, Rwanda.

**Methods:**

a cross-sectional design was conducted in October 2021. The sample was 345 participants selected by cluster simple random sampling from a targeted population of 1426 women. The interviews were conducted, and structured questionnaires were filled out before entering and analyzing data into Statistical Package Social Sciences (SPSS). Descriptive statistics analysis was used to determine frequencies and percentages, while multivariate seconded the bivariate regression analysis determine the factors associated to dual contraception by odds ratio with 95% CI.

**Results:**

the mean age of interviewees was 35.59 years and the majority of them were married women (60.9%). The dual contraception rate was 40%. The multivariate analysis of factors associated with dual contraception revealed that single women were most likely (AOR=38.123, 95% CI: 6.575-221.040, p<0.001) to use combinations of condoms and other methods. The odds of utilizing dual contraceptive methods were 7.347 times (AOR=7.347, 95% CI: 0.936-57.671, p=0.049) higher among women whose partners were casual laborers. Women who did not desire to have a baby were most likely (AOR=3.990, 95% CI: 1.796-8.865, p=0.001) to utilize dual contraception. The odds of utilizing dual methods were 5.634 times (AOR=5.634, 95% CI: 2.277-13.939, p<0.001) higher among women whose sexual partners did not desire a baby compared to those whose partners did. The odds of using dual methods were 1.354 times (AOR=1.354, 95% CI: 0.705-2.602) higher among women who disclosed their HIV status to their sexual partners compared to those who did not. The odds of using dual contraception were 5.526 times (AOR=5.526, 95% CI: 2.186-13.968, p<0.001) higher among women who were in HIV program for more than five years compared to those who were in the program for one year or less.

**Conclusion:**

the rate of dual contraception in this area is still low according to World Health Organization (WHO) recommendation and strategies to increase it are of paramount importance to be put in place by the Ministry of Health through Rwanda Biomedical Center, health facilities and partners in terms of training, health education, availability of dual methods at the level of the health system and men involvement in family planning.

## Introduction

One of the major problems relating to public health in countries with low and middle income is rapid population growth that is not proportional to the wealth within them. The only strategy which is highly cost-effective to control the fertility rate in developing countries is effective and consistent family planning, which is of paramount importance to deciding when and how many children a couple will possess [1]. World Health Organization (WHO) recommended that every single sexually active woman should use any Family planning (FP) method combined with a condom to stop new HIV infection and prevent sexually transmitted infections (STI) [2]. Dual contraception is the combination of two contraceptives where one of which is a condom to doubly protect against unwanted pregnancies, HIV, and sexually transmitted infections within sexual partners. The dual protection in women enrolled in HIV program under antiretroviral treatment (ART) and their partners protect against sexually transmitted infections (STIs), limits the risk of new strains of Human Immunodeficiency Virus (HIV) and prevents unplanned pregnancies and of course limitation of vertical transmission of HIV [3]. Worldwide, 40% of maternal deaths can be avoided by family planning programs implementation and utilization by the beneficiaries. By 2015, the worldwide percentage of FP methods utilization among married or cohabiting women aged 15-49 years was 64%, and it was low in Africa where the percentage was only 33%. Globally, 225 million women would like to not get pregnant but were not able to have safe and effective family planning methods [4]. In 2019, the modern contraceptive prevalence rate among women all over the world stood at 91.3% and the low rates were observed in low and middle-income countries (LMICs) compared to the high rates in developed countries. The prevalence of using modern contraception in the USA was 90.7%, 96.8% for Australia, 96.6% for Brazil, 98.1 for Germany. In Eastern Africa, the modern contraceptive prevalence rates were 90.7% for Uganda, 90.6% for Rwanda, 97.1% for Kenya, 94.4% for South Sudan, 87.4% for Burundi and 86.3% for Tanzania [5].

Despite efforts put in place by countries in Prevention of mother-to-child transmission (PMTCT), there are still a non-neglected risk of HIV infection as the outcome at 24^th^ month of life of the child in developing countries including Rwanda with low utilization of both single and dual family planning methods. In Rwanda, 2% of exposed infants born from HIV-positive mothers are confirmed positive at 24^th^ month of life [6]. Women who use the dual contraceptive in reproductive age on antiretroviral followed up in health facilities in sub-Saharan are still making a small number where the rate are still low. A study done in Ethiopia in December 2018 showed that dual contraceptive utilization was low in three different regions; 19.8 percent in Southwest Ethiopia, 13.8 percent in Tigray, 13.2 percent in Gondar [7]. Globally, 600,000 women died of complications due to pregnancies among more than 2 million of women on antiretroviral treatment who fall pregnant each year secondary to low utilization of dual [8]. The dual contraception associated factors varied from individual study to another and the most common among women in reproductive age on ART for previous studies showed odds with association to outcome variable along the 95% CI and significance by p-value analysis were age, religion, marital status, education of the women, number of children who are still alive, couple´s desire to have a child in the future, the status of HIV for the partner, HIV status disclosure and the duration in HIV program [9,10]. The contraceptive prevalence rate in Rwanda has increased where it was 14%, 17%, 51%, 54% and 64% according to Robinvale District Health Service (RDHS) 2000, RDHS2005, RDHS 2010, RDHS 2015 and RDHS 2020 respectively. However, the fertility rate still high and it has varied with a small change from 6.1, 5.5, 4.6, 4.2, and 4.1 for the above five last surveys in Rwanda within 20 last years. Even though the percentage is low but HIV/AIDS confirmed children would face a range of problems of starting drugs at low age towards the course of their whole life associated with several psychosocial issues. The mean number of sexual partners in a lifetime for a woman in the Eastern province of Rwanda was 1.7%.

The percentage those who had sex with other people who were not their husbands was 8.7%, 1% of them said they had two or more sexual partners and only 38.5% of women had used a condom. It was also revealed that the mean of number of sexual partners in lifetime for a man in the same province was 2.8%. The percentage of men who had two or more sexual partners was 6.2% and 12% of men said, they had sexual intercourse with other women who were not their wives and only 30.4% used condoms. The trends regarding FP methods in Eastern Province where Kayonza District is located revealed the increase along with time. The contraceptive prevalence rate was 9%, 18.9%, 46% and 53.9% in East of Rwanda according to Rwanda Demographic and Health Survey done in 2000 [11], Rwanda Demographic and Health Survey done in 2005 [12], Rwanda Demographic and Health Survey done in 2010 [13], Rwanda Demographic and Health Survey done in 2015 [14] respectively. The last Rwanda Demographic and Health Survey done in 2020 showed that the contraceptive prevalence rate in the same province where Kayonza District is located was 66.1% with the fertility rate that stood at 4.1 and remained high [15]. Using condoms among women in reproductive age helps to prevent the infection caused by HIV, STIs, unplanned pregnancy, but when it is used incorrectly and inconsistently, it fails at 14% and women get pregnant during the first year of poor utilization [16]. In Kayonza District, 44.7% of people on antiretroviral therapy are women aged between 15-49 years. This high percentage enabled the researcher to conduct a study revealing the situation of dual method utilization among these women aged 15-49 years and taking ARVs by computing the proportion, types of combined methods used identifying its associated factors. In Rwanda, there is no single publication on dual contraceptive use and its associated factors among women in reproductive age on ART. To fill the above said gap, the present study had two objectives: one is to assess dual contraception prevalence and the second is to identify the dual contraception associated factors among these women in Kayonza District.

## Methods

**Study design:** a cross-sectional study with a quantitative approach was conducted in October 2021 to determine the rate of dual contraceptive use and identify its associated factors among women aged 15-49 years in Kayonza District, Eastern Province of Rwanda.

**Study setting:** the study was conducted in the health centers of Kayonza District. This district is one of the seven districts that compose the Eastern Province of Rwanda. Our country is composed of 30 districts that are grouped in four provinces (East, North, West, South, and Kigali) and Kigali City. Rwanda is located in East Africa with four neighboring countries: Uganda in the north, Burundi in the South, DR Congo in the West, and Tanzania in the East.

**Target population and sample size:** the target population of the present study included women who were aged 15-49 years on antiretroviral therapy, enrolled, and followed up in health centers located in Kayonza District, Eastern Province of Rwanda. The calculation of the present study sample size was done to determine the participants who were interviewed by trained data collectors to fill the structured questionnaires that provided data for analysis after being cleaned and entered into SPSS 21 software. The number of participants was determined using Yamane Taro´s formula: where n is the sample size, N is the population, 1: it is one, it is not a letter, e: margin of error (0.05)


$$n=\frac{N}{1+N{\left(e\right)}^{2}}$$

$$n=\frac{1426}{1+1426{\left(0.05\right)}^{2}}=313$$

The sample will be n=1,426/ [1+1,426(0.05)2] =313. The sample was made of 313 participants plus an increment of 10%. Therefore, the sample was made of 345 participants. The sample size for each health center was proportionally calculated and was in function of the number of women who are in reproductive age and taking ART there. To select the study participants, cluster simple random sampling was applied where health centers were considered as clusters. Women who were aged 15-49 years on ART and who were already pregnant or who were lost to follow-up within three last months were excluded from the study.

**Study variables:** the dependent variable for our study was dual contraceptive use and the independent variables were grouped into four: the first comprised sociodemographic and economic characteristics of the participants (age, religion, marital status, patient´s education, partner´s education, patient´s occupation, partner occupation and level of income in Rwanda-ubudehe category). The second was made of sexual and reproductive health factors (number of children alive, sexual partners´ number, partner HIV status, desire of the patient to have a baby, and desire of the sexual partner to have a baby). The third was a factor related to FP package provision by health care providers (post-test counseling including dual contraception) and the last was made of factors related to the participant´s past medical history (HIV status disclosure to partner, duration of follow-up by HIV program, patient´s previous treatment of STIs and unplanned pregnancy in the past).

**Data collection instruments and procedures:** for every single participant, a structured questionnaire was completely filled. The data collectors interviewed the participants according to predefined questions on study variables and provide the possible answers so that the women selected the convenient ones for some questions on one side while on the second side, questions were asked and the women gave the answers themselves. The trained data collectors met the participants in different health centers to gather data relating to the questionnaire. They took the patients in a comfortable place, and then interviews were conducted to fill out the structured questionnaire.

**Data analysis:** after collecting the data from respondents through a questionnaire, they were entered and analyzed with SPSS 21 software. To determine the rate of dual contraceptive use, and the current types of dual contraceptive methods, descriptive statistics analysis was used. The odds ratios with 95% CI were applied to establish the factors modifying positively or negatively the dual contraceptive utilization by women in reproductive age on ART in Kayonza District. The findings of the study were considered significant for a p-value <0.05. Tables and text were used in a Word document to present the findings of the study.

**Ethical consideration:** prior to data collection, the study was approved by Mount Kenya University Rwanda, the School of Postgraduate Studies and Kayonza District approved and provided the permission to conduct the research. Every study participant signed a consent form before interviewing her. The participants were assured that their data must stay confidential and must be kept without their names. The names of the patients helped to identify them for an interview, but they have not to appear on the questionnaire.

## Results

**Socio-economic and demographic analysis:** the findings of the present study in terms of sociodemographic and economic status revealed that 26.7% of all study participants are currently being followed up at Kabarondo HC for ART and FP. Of the 56.5% were aged 35 years and above, the mean age was 35.59 years, the minimum and maximum ages were 15 and 49 respectively, 48.1% were Catholic, and 60.9% were married. According to the level of education for study participants and that of their partners, the majority of patients and partners had the primary level of education where 65.5% of participants finished their primary education and 69.3% of their partners had the same education. It was revealed that the occupation of the interviewees and their partners was mainly farming; 72.8% of the interviewees were farmers while 65.2% of their partners were declared farmers too and only 2.0% of the patients were working for government and Non-Governmental Organizations (NGOs), while 2.3% of their partners were employed by government or NGOs. The study results showed that most of the interviewees were found in ubudehe categories 3 and 4 (50.4%) among four categories that define levels of income in Rwanda (Table 1).

**Table 1:** socio-economic and demographic characteristics of respondents

Variables	Frequency (n=345)	Percentage (%)
**Health facility**		
Rwinkwavu HC	69	20.0
Rutare HC	15	4.3
Ruramira HC	29	8.4
Nyamirama HC	50	17.4
Ndego HC	24	7.0
Karama HC	22	6.4
Kabarondo HC	92	26.7
Cyarubare HC	34	9.9
**Age (years)**		
15-24	40	11.6
25-34	110	31.9
35-49	195	56.5
**Religion**		
Catholic	166	48.1
Protestant	127	36.8
Islam	10	2.9
No religion	42	12.2
**Marital status**		
Married	210	60.9
Single	56	16.2
Separated	44	12.2
Divorced/widowed	35	10.1
**Patient's level of education**		
Uneducated	91	26
Primary	226	65.5
Secondary	28	8.1
**Partner's level of education**		
Uneducated	80	23.2
Primary	239	69.3
Secondary	26	7.5
**Occupation of the patient**		
Housewife	31	9.0
Farmer	251	72.8
Merchant	14	4.1
Employed	7	2.0
Casual laborer	30	8.7
Others	12	3.5
**Partner's occupation**		
No occupation	28	8.1
Farmer	225	65.2
Merchant	16	4.6
Employed	8	2.3
Casual laborer	34	9.9
Others	34	9.9
**Ubudehe category (level of income in Rwanda)**		
Ubudehe category 1	46	13.3
Ubudehe category 2	125	36.2
Ubudehe category 3 and 4	174	50.4

**Descriptive statistics analysis to determine the dual contraceptive rate:** the sample was made of 345 interviewees. All participated, of the 138 women used dual contraception, 65 women used single method and 142 did not use any method neither single nor dual. The dual contraception rate in Kayonza District was 40%. The types of dual methods used in this district were condoms + implants (25.2%) that were highly used, followed by condoms+ injectable (7.0%), condoms + pills (5.8%), condoms + intrauterine device (IUD) (0.9%), condoms + vasectomy (0.9%) and lastly condoms + Bilateral tubal ligation (0.3%). The findings showed also that Rwinkwavu HC was found as the health facility where many of the interviewees were highly utilizing dual methods (11.9%) while Ruramira HC showed only 1.4% of them (Table 2).

**Table 2:** dual contraceptive utilization rate among women aged 15-49 years on antiretroviral therapy in Kayonza District, Rwanda, 2021

Variables	Frequency	Percentage (%)
**Current dual contraceptive utilization (n=345)**		
**Dual contraception users**	**138**	**40.0**
Not users	207	60.0
**Dual contraception rate by types of combinations (n=138)**		
Condoms + pills	20	5.8
Condoms + injectables	24	7.0
Condoms + implants	87	25.2
Condoms + intra-uterine device	3	0.9
Condoms + bilateral tubal ligation	1	0.3
Condoms + vasectomy	3	0.9
**Women aged 15-49 years on antiretroviral therapy using dual methods by health center in Kayonza District (n=138)**		
Rwinkwavu HC	41	11.9
Rutare HC	8	2.3
Ruramira HC	5	1.4
Nyamirama HC	30	8.7
Ndego HC	12	3.5
Karama HC	9	2.6
Kabarondo HC	24	7.0
Cyarubare HC	9	2.6

**Bivariate logistic regression analysis:** the findings from the crude analysis revealed that women who were married were most likely (OR=2.943, 95% CI: [1.517-5.710], p=0.001) to utilize dual contraceptives when taking ART than widowed women. The OR of utilizing dual contraceptives were 2.133 times (OR=2.133, 95% CI: [1.256-3.623], p=0.005) higher among women with primary education compared to those who were uneducated. The odds of using dual contraception were 2.753 times (OR=2.753[1.558-5.004], p=0.001) higher among interviewees´ sexual partners with primary education compared to those without any level of education. Women whose sexual partners where farmers were most likely (OR=1.575, 95%CI: [0.734-3.348]) to choose combinations of condoms and other family planning methods than those with other professions or occupations. The odds of utilizing dual methods were higher (OR=4.892, 95% CI: [2.012-9.989], p=<0.001) among women who were registered in Ubudehe category 3 and 4 (level three and four of income in Rwanda) than other remaining categories. The odds of utilizing dual contraception were 12.5 times (OR=12.5, 95% CI: [6.832-22.870], p<0.001) higher among women who did not desire of having a baby in the future compared to those who desired to have a baby even though they were HIV-positive. The odds of using dual contraceptive methods were 16.0 times (OR=16.0, 95% CI: [7.979-32.204], p<0.001) higher among women who said that their sexual partners did not desire to have a baby compared to those who desired a baby even though they were HIV-positive. The findings showed that women who disclosed their HIV status to their sexual partners were most likely (OR=1.6, 95% CI: [1.045-2.755], p=0.032) to use dual contraception than those who did not disclose their status to partners. The duration of staying for a long time in HIV program was associated with the use of dual contraceptive methods among women in reproductive age on ART in Kayonza District; the OR of using dual methods were 1.654 times ( OR=1.654, 95% CI: [0.582-4.700]) higher among women who were in HIV program for more than eight years compared those with one year or less in a program which means that the findings showed that the more the women are in the program for a long time, the more they will utilize the dual contraception (Table 3).

**Table 3:** bivariate and multivariate logistic regression analysis of factors associated to the use of dual contraceptives among women aged 15-49 years on antiretroviral therapy in Kayonza District, Rwanda, 2021

Variables	Crude OR [95% CI]	P-value	AOR [95% CI]	P-value
**Patient marital status**				
Married	2.943 [1.517-5.710]	0.001	9.534 [2.100-32.27900]	<0.001
Single	0.644 [0.270-1.536]	0.321	38.123 [6.575-221.040]	<0.001
Separated	2.793 [0.893-8.732]	0.077	37.173 [7.020-196.845]	<0.001
Divorced/widowed	Ref		Ref	
**Level of education of the patient**				
Uneducated	Ref		Ref	
Primary	2.133 [1.256-3.623]	0.005	0.045 [0.019-1.090]	0.998
Secondary +	1.834 [0.611-5.490]	0.009	0.032 [0.012-1.005]	0.998
**Level of education of the patient's partner**				
Uneducated	Ref		Ref	
Primary	2.753 [1.558-5.004]	0.001	0.067 [0.028-1.210]	0.996
Secondary +	2.492 [0.878-6.475]	0.068	0.051[0.022-1.231]	0.996
**Partner's occupation**				
No occupation	Ref		Ref	
Farmer	1.575 [0.734-3.348]	0.235	2.752 [0.574-13.194]	0.205
Merchant	0.833 [0.234-2.967]	0.778	5.607 [0.441-71.344]	0.184
Employed	0.796 [0.119-4.389]	0.746	0.150 [0.083-1.0110]	0.998
Casual laborer	0.393 [0.127-1.214]	0.104	7.347 [0.936-57.671]	0.049
Others	0.762 [0.238-2.161]	0.574	2.250 [0.255-19.894]	0.466
**Ubudehe category (level of income in Rwanda)**				
Ubudehe category 1	Ref		Ref	
Ubudehe category 2	0.877 [0.394-1.952]	0.747	0.123 [0.007-1.003]	0.994
Ubudehe cat. 3 and 4	4.892 [2.012-9.989]	0.021	0.190 [0.021-0.982]	0.979
**Desire of the respondent in planning to have a baby**				
Yes	Ref		Ref	
No	12.5 [6.832-22.870]	<0.001	3.990 [1.796-8.865]	0.001
**Desire of sexual partner to have a baby**				
Yes	Ref		Ref	
No	16.0 [7.979 32.204]	<0.001	5.634 [2.277-13.939]	<0.001
**HIV status disclosure to partner**				
Yes	1.697 [1.045-2.755]	0.032	1.354 [0.705-2.602]	0.363
No	Ref		Ref	
**Duration of follow-up by HIV program (in years)**				
0-1	Ref		Ref	
2-3	0.323 [0.118-0.885]	0.028	7.608 [0.904-64.062]	0.062
4-5	0.483 [0.157-1.484]	0.204	3.020 [1.109-8.2250]	0.031
6-7	0.714 [0.241-2.119]	0.544	5.526 [2.186-13.968]	<0.001
8-9	1.654 [0.582-4.700]	0.345	2.552 [0.958-6.7970]	0.061
10+	1.105 [0.363-3.364]	0.860	1.096 [0.433-2.7750]	0.847

**Multivariate logistic regression analysis:** among nine factors found significant in bivariate analysis, six were concluded by the multivariate analysis: Marital status, sexual partner´s occupation, patient desire to have a baby, partner desire to have a baby, disclosure of patient HIV status to partner and the duration in years of follow-up in HIV program at the health facility. The single women were most likely (AOR=38.123, 95% CI: [6.575-221.040], p<0.001) to use combinations of condoms and other methods than other women in the study. The odds of utilizing dual contraceptive methods were 7.347 times (AOR=7.347, 95% CI: [0.936-57.671], p=0.049) higher among women whose partners are casual laborers than in other partners´ occupations. The study findings revealed that women who did not desire to have a baby were most likely (AOR=3.990, 95% CI: [1.796-8.865], p=0.001) to utilize dual contraception than those who desired to have a baby. The odds of utilizing dual methods were 5.634 times (AOR=5.634, 95% CI: [2.277-13.939], p<0.001) higher among women whose sexual partners did not desire a baby compared to those whose partners desired to have a baby even though they were HIV-positive. The results showed also that the odds of using dual methods were 1.354 times (AOR=1.354, 95% CI: [0.705-2.602]) higher among women who disclosed their HIV status to their sexual partners compared to those who did not. The odds of using dual contraception were 5.526 times (AOR=5.526, 95% CI: [2.186-13.968], p<0.001) higher among women who were in HIV program for more than five years compared to those who were in the program for one year or less (Table 3).

## Discussion

The findings from this research revealed that a majority of study participants (56.6%) were aged 35 years and above and most of them are married (60.9%). According to the level of education for study participants and that of their partners, the majority of patients and partners had the primary level of education where 65.5% of participants finished their primary education and 69.3% of their partners had the same level of education. Seventy-two point eight (72.8%) of interviewees were farmers and 65.2% of interviewees´ partners were farmers too. These findings were in line with results from previous studies: the studies conducted in Borena District, Southern Ethiopia, in six hospitals in Thailand, in Bungoma county of Kenya, in Northern Ethiopia [17], in South East Nigeria [18], and in Kilimanjaro region, Northern Tanzania [19], showed the similar results where the study participants were aged 35 years or more, a big number of them were also married and most participants were of primary education only. Other previous studies such the ones conducted in Ibadan of Nigeria, in South East Nigeria, in the USA [20] revealed the contrast in fact that the findings showed that most of the interviewees were aged less than 35 years. Other studies such the ones conducted in South Africa [21] and in Lusaka of Zambia [22] showed the contrast in level of education where most of the study participants had completed the secondary education or above. The first objective of the study was to estimate the dual contraceptive utilization rate among women in reproductive age on ART in Kayonza District. The results revealed that in this district, 40% of women interviewed were using dual contraceptives to prevent both pregnancy, HIV and STIs and their related consequences. The dual contraception rate of the present study was in line with findings found for a study conducted in Ibadan, Nigeria that stood at 41.5%. The findings of the this study were low compared to the rates estimates in a study done in USA that showed that the utilization of dual contraceptives stood at 58% and the one carried out in Uganda with 58% too [23]. However, the rate of dual contraception in Kayonza District was higher than those found in studies conducted in Borena of Ethiopia with 19.4% and Northern Ethiopia with 15.7%.

The idea for all people living with HIV/AIDS is to utilize dual contraceptive method as recommended by WHO. The findings of all of these studies were still low as they were not yet still around a hundred percent. This may be due to inaccessibility of FP methods, shortage of trained health care providers and cultural factors that differ from a country to another with different health system strengthening strategies. The results of this study revealed that the combination condoms + implants were highly used (25.2%), followed by condoms + injectable (7.0%), condoms + pills (5.8%), condoms + IUD (0.9%), condoms + vasectomy (0.9%) and lastly condoms + Bilateral Tubal Ligation (BTL) (0.3%). The difference between these findings and other previous studies conducted in different countries was clear and explained by the results which were different by considering the top two dual methods each study: the top two types of dual contraceptives methods used found in Borena District of Ethiopia were condoms + injectable followed by condoms + pills, in six hospitals of Thailand the combinations of condoms + sterilization were followed by condoms + oral pills, in Bungoma county of Kenya the combinations condoms + injectable came before condoms + implants and in South East Nigeria condoms + injectable came before condoms + oral pills. The most common combinations were condoms + oral pills were found in all the above-mentioned studies. The results of this study showed a low level of utilization of condoms plus long acting methods where the condoms + Vasectomy and condoms + Bilateral Tubal Ligation while in Thailand the first used combination was condoms + sterilization for male and female. This explained the risk of possible lost to follow-up cases, contraception failure, and related consequences in Kayonza District. The differences among chosen types of dual methods may vary according to availability of contraceptive methods and the presence of trained health care providers associated to consistent counseling and health education at all times in health facilities. The second and last objective of this study was to identify factors associated to dual contraceptive method's utilization among women in reproductive age on ART in Kayonza District.

The multivariate regression analysis showed that marital status, sexual partner´s occupation, desire of the patient to have a baby, desire of partner to have a baby, disclosing of patient HIV status to partner and the duration in years of follow-up in HIV program at health facility were factors associated to the utilization of dual contraceptive methods in this district. The findings from multivariate analysis revealed that single women were most likely to choose dual contraception (AOR=38.123, 95% CI: .575-221.040], p<0.001). This finding was similar and supported by the studies conducted in Ibadan, Nigeria and Northern Ethiopia and this may be due to the fact that single women avoided the risk of unplanned pregnancies and STIs with associated consequences and complications as they were living alone. In the contrary, studies conducted in Borena District of Ethiopia and Thailand revealed that most of women aged 15-49 years on ART who used dual methods were married and this because married women found time to discuss with their partners about desire to get pregnant in the future after being infected by HIV. The women whose sexual partners were casual laborers in this study had an increased likelihood of using dual contraceptives (AOR=7.347, 95% CI: [0.936-57.671], p=0.049) compared to other occupations. This was similar to a study conducted in Ethiopia in Borena District of Ethiopia. The likelihood of utilizing dual contraception in this study was significantly associated with patient without desire to have child in the future after being diagnosed HIV+ (AOR=3.990, 95% CI: [1.796-8.865], p=0.001) compared to those who desired have children. This was similar and supported by a study conducted in Borena District of Ethiopia. Most of sexual partners did not desire to have children in the future according this research (AOR=5.634, 95% CI: [2.277-13.939], p<0.001) and this was opposing to the results of the same study conducted in Borena District, Ethiopia where the sexual partners of study interviewees were most likely to desire in having babies. Women who disclosed their HIV status to their respective partners mostly used dual contraceptives (AOR=1.354, 95% CI: [0.705-2.602]) than those who secretly kept their diagnosis.

This finding was supported by a study conducted in Bungoma county of Kenya and Northern Ethiopia but the one carried out in Borena District of Ethiopia [3] revealed that the likelihood of using dual methods was decreased for women who disclosed their status to their sexual partners. The study findings showed that most women who were enrolled for dual contraceptives were the ones who have been in HIV programs for a long time. The odds of using dual contraception were 5.526 times (AOR=5.526, 95% CI: [186-13.968], p<0.001) higher among women who were in HIV program for more than five years compared to those who were in the program for one year or less. This was similar to the findings of a study conducted in Kilimanjaro region, Northern Tanzania. The first limitation was that this study was carried out under cross-sectional design that evaluated the factors associated to the outcome variable simultaneously and of course, we cannot establish the causal association between dependent and independent variables. Secondly, the study was conducted in health centers that are located in Kayonza District; no generalization of findings of this study to the whole country as only one was covered among thirty districts of Rwanda.

## Conclusion

The dual contraception rate among women in reproductive age on ART in Kayonza District was still low compared to the recommendation of WHO even it was greater than the rates revealed by other studies in some countries; this requires to go on a long journey to increase the dual contraceptives utilization to prevent HIV transmission from mother to child in the womb, unplanned pregnancies and STIs. The factors associated to dual contraception utilization were marital status, sexual partner´s occupation, patient desire to have a baby, partner desire to have a baby, disclosure of patient HIV status to partner and the duration in HIV program after diagnosis of HIV-positive infection. To increase the utilization of dual contraceptives among women sexually active who take ART, some activities should be revised and put into action and they should be related to availability of FP methods at all levels without stock out, training of new and existing health care providers at all levels of Rwanda Health System on counseling and involvement of men in choice and adoption of dual contraceptives among people with HIV.

### What is known about this topic

- The dual contraceptive rate among women aged 15-49 years on antiretroviral therapy in different countries other than Rwanda;
- Factors associated to dual contraceptive use among women aged 15-49 years on antiretroviral therapy in different countries other than Rwanda.

### What this study adds

- Dual contraceptive rate and types of dual contraceptive methods among women aged 15-49 years on antiretroviral therapy Kayonza District, Eastern Province of Rwanda;
- Factors associated to dual contraception among women aged 15-49 years on antiretroviral therapy in Kayonza District, Eastern Province of Rwanda;
- The dual contraceptive rate was low in Kayonza District of Rwanda, as it was in low and middle-income countries.
